# Mechanical Circulatory Support with iVAC2L During High-Risk Percutaneous Coronary Intervention: A Case Report

**DOI:** 10.3390/jcm15187322

**Published:** 2026-09-21

**Authors:** Gerda Sibirkstyte, Mindaugas Barauskas, Martynas Jurenas, Ramunas Unikas, Marcelo B. Bastos, Chrisna de Wet Breukelen

**Affiliations:** 1 Faculty of Medicine, Medical Academy, Lithuanian University of Health Sciences, 44307 Kaunas, Lithuania; gerda.sibirkstyte@lsmu.lt (G.S.);; 2 Department of Interventional Cardiology, Erasmus University Medical Center, Dr. Molewaterplein 40, 3015 GD Rotterdam, The Netherlands

**Keywords:** coronary artery disease, high-risk percutaneous coronary intervention, mechanical circulatory support, iVAC2L, left main coronary artery, case report

## Abstract

**Background:** High-risk percutaneous coronary intervention (HR-PCI) in patients with severe left ventricular (LV) systolic dysfunction and complex coronary artery disease may be complicated by abrupt intraprocedural hemodynamic deterioration. Temporary mechanical circulatory support may be considered after individualized Heart Team assessment; however, clinical experience with the pulsatile iVAC2L device remains limited. **Case Presentation:** An 84-year-old man presented with inferior ST-segment-elevation myocardial infarction, acute decompensated heart failure, pulmonary edema, and an LV ejection fraction of 10–15%. Coronary angiography (CAG) showed a presumed chronic total occlusion of the right coronary artery (RCA) and critical disease of the left main (LMCA), left anterior descending (LAD), and left circumflex (LCx) arteries. Preparation for coronary artery bypass grafting was initiated, but clinical deterioration prompted PCI. The first attempt was aborted after cardiac arrest during coronary wiring. Following stabilization and repeat Heart Team assessment, iVAC2L-supported HR-PCI was performed. During 60 min of active pump support, five stents were implanted in the LMCA, LAD, and LCx. The patient remained conscious and hemodynamically stable without vasopressors. LV ejection fraction was approximately 20% at discharge after six hospital days, and the patient was transferred to inpatient rehabilitation. **Conclusions:** In this selected patient, iVAC2L-supported HR-PCI was technically feasible and was associated with maintained intraprocedural hemodynamic stability. A single case cannot establish comparative safety or efficacy; prospective studies are required to define patient selection, procedural protocols, and clinically meaningful outcomes.

## 1. Introduction

Patients referred for complex PCI increasingly present with adverse clinical and anatomical characteristics. Advanced age, chronic kidney disease, peripheral arterial disease, previous myocardial infarction or stroke, and markedly impaired LV function reduce physiological reserve, whereas unprotected left main disease, multivessel disease, long lesions, atherectomy, and multiple arterial access sites increase procedural complexity. The BCIS-CHIP score incorporates several of these features and can assist in stratifying the risk of major adverse cardiac and cerebrovascular events associated with complex PCI [1].

During HR-PCI, transient interruption of coronary flow, ischemia, arrhythmia, or changes in preload and afterload may be poorly tolerated in patients with severely impaired LV systolic function. Temporary mechanical circulatory support (MCS) may therefore be considered to maintain systemic perfusion during revascularization [2,3,4]. However, there is no universally accepted definition of HR-PCI or validated threshold for prophylactic MCS. Device selection should consider hemodynamic status, coronary anatomy, ventricular function, vascular access, bleeding risk, procedural complexity, device availability, and operator experience [3,4].

Clinical experience with iVAC2L-supported PCI remains limited to small observational studies and case reports. Samol et al. described iVAC2L-supported PCI in an 80-year-old patient with preserved LV ejection fraction and recurrent severe coronary vasospasm [5]. Another case report described multivessel LAD, LCx, and RCA revascularization following TAVI, complicated by respiratory discomfort and increased LVEDP during support [6]. Nicolau et al. reported iVAC2L-supported LMCA, LAD, and LCx PCI in an 81-year-old patient with severe calcified multivessel disease and reduced LV ejection fraction (34%) following Heart Team evaluation [7].

We present an 84-year-old patient with STEMI, severely impaired LV systolic function (15%), and multivessel coronary disease, in whom an initial unsupported PCI attempt was complicated by cardiac arrest. Following repeat Heart Team evaluation, extensive LMCA, LAD, and LCx revascularization with planned temporary MCS was performed. The novelty of this case lies in its exceptional clinical, anatomical, and procedural complexity rather than in the use of iVAC2L itself. It highlights the importance of repeated Heart Team assessment when procedural risk changes substantially after an initially unsuccessful intervention, allowing reconsideration of the revascularization strategy and need for temporary MCS.

This case report was prepared in accordance with the CARE (CAseREport) guideline, and a completed CARE checklist is provided as Supplementary File S1.

## 2. Case Presentation

An 84-year-old man was transferred from a regional hospital to the intensive cardiac care unit (ICCU) of a tertiary university hospital because of inferior STEMI complicated by acute decompensated heart failure, pulmonary edema, and severe LV systolic dysfunction. His medical history included arterial hypertension, chronic heart failure, previous surgery for a penetrating chest injury, and stage 3 chronic kidney disease. Before admission, he was taking perindopril, indapamide, spironolactone, and ranolazine. On transfer, he was conscious and cooperative, with blood pressure 140/86 mmHg, heart rate 105 beats/min, respiratory rate 20 breaths/min, and oxygen saturation 91% while receiving oxygen at 12 L/min. Numerous bilateral pulmonary crackles were present, and the clinical status was classified as Killip class III.

**Figure 1:**
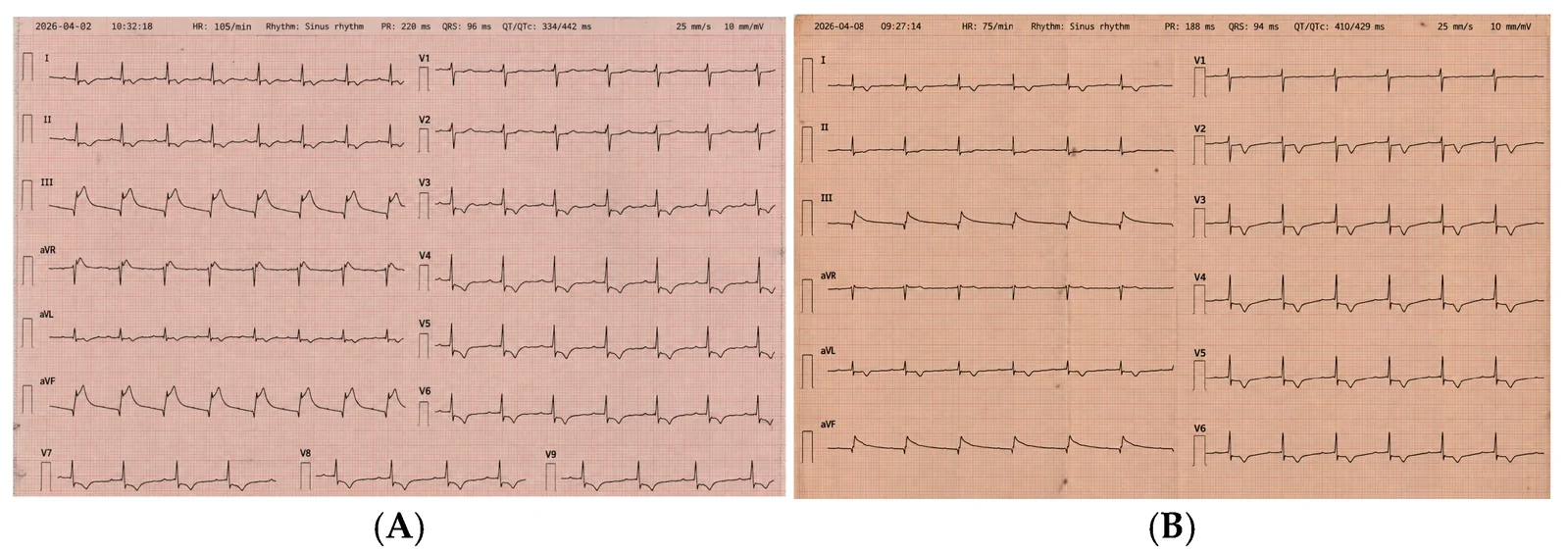
Electrocardiogram: (**A**) The initial ECG demonstrated sinus rhythm at 105 beats/min with first-degree atrioventricular block, ST-segment elevation in leads III, aVF, and aVR, and ST-segment depression in leads I, II, aVL, V4–V6, and posterior leads. (**B**) The ECG performed on the last day of hospitalization showed sinus rhythm at 75 beats/min, persistent ST-segment elevation in leads III and aVF, and T-wave inversion in leads I, aVL, and V2–V6.

The initial ECG demonstrated an inferior STEMI pattern; an ECG obtained on the last day of hospitalization is also presented in the same figure to illustrate the subsequent electrocardiographic evolution (Figure 1). Initial laboratory results and their changes over time are presented below (Table 1).

Transthoracic echocardiography showed fibrodegenerative aortic-valve changes without significant functional impairment, mild mitral regurgitation, a dilated LV with eccentric hypertrophy, and severely reduced global LV systolic function. The basal and mid interventricular septal segments contracted slightly better than the remaining myocardium. Visual LVEF was 10–15%; the apex and lateral and inferior walls were akinetic, and the anterior wall was markedly hypokinetic. Grade I LV diastolic dysfunction was present. The right atrium and right ventricle were not dilated, longitudinal right ventricular function was preserved, mild tricuspid regurgitation was present, and pulmonary artery pressure was estimated to be moderately elevated.

**Figure 2:**
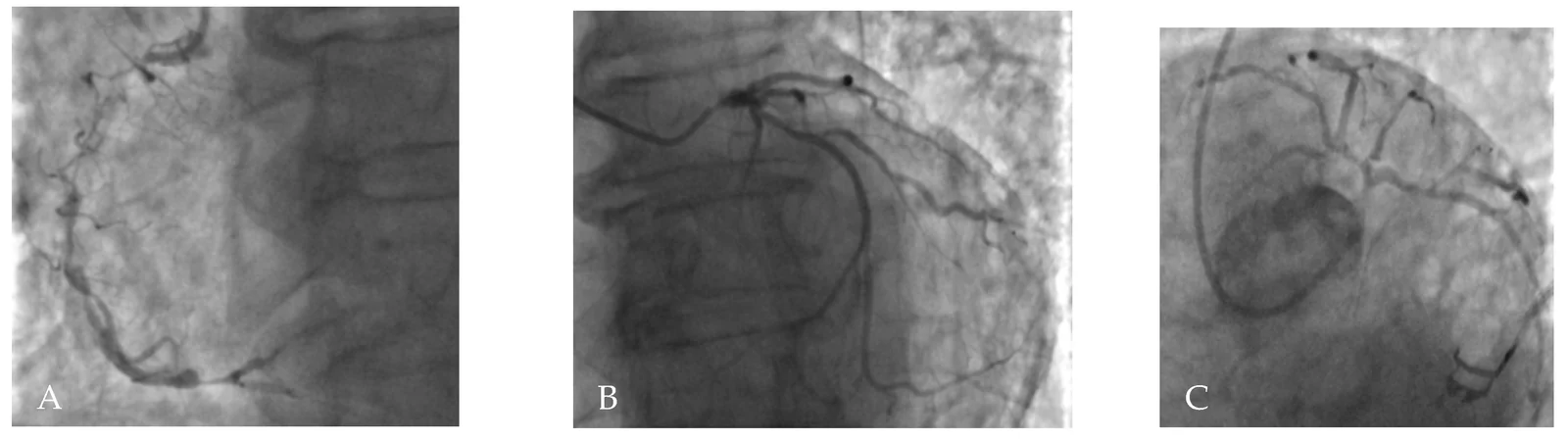
Baseline CAG demonstrating complex multivessel coronary artery disease: (**A**) Presumed CTO of the RCA with well-developed left-to-right collateral circulation and complete retrograde filling of the distal epicardial RCA (Rentrop grade 3). (**B**) Critical 99% LMCA stenosis with stenosis of the LAD and LCx. (**C**) Critical 99% LAD stenosis and 70% LCx stenosis. The angiographic findings demonstrated critical left-coronary disease supplying a large myocardial territory and providing collateral perfusion to the occluded RCA.

Initial CAG demonstrated a presumed chronic total occlusion (CTO) of the RCA together with critical multivessel left-coronary disease, including 99% LMCA stenosis, 99% LAD stenosis, and 70% LCx stenosis (Figure 2). The RCA occlusion was considered likely chronic based on well-developed left-to-right collateral circulation from the LAD and LCx, with complete retrograde filling of the distal epicardial RCA, corresponding to Rentrop grade 3 collateral flow. However, in the absence of prior CAG documenting the duration of the occlusion, it was classified as a probable rather than definitively established CTO. Given the dependence of the inferior territory on collateral perfusion from the severely diseased left coronary system, the working pathophysiological interpretation was that critical LMCA, LAD, and LCx disease reduced donor-artery pressure and compromised collateral flow to the RCA territory, thereby precipitating acute inferior-wall ischemia and inferior ST-segment elevation despite the apparently chronic RCA occlusion.

After the initial CAG and interventional cardiology assessment, the patient was started on preparation for CABG. Over the following several hours, however, pulmonary edema progressed, chest pain recurred, and repeated ventricular tachycardia was observed on cardiac monitoring. A multidisciplinary discussion involving cardiac surgeons, intensive-care physicians, and the interventional cardiologist concluded that the operative risk was prohibitively high; therefore, PCI was attempted through right radial arterial access. Shortly after a guidewire was advanced into the LCx, progressive bradycardia occurred, and the patient lost consciousness. The guidewire was immediately withdrawn, and external chest compressions were initiated, with return of spontaneous circulation after approximately 30 s. Given the LCx stenosis and the immediate temporal relationship between guidewire insertion and hemodynamic collapse, transient further compromise of coronary blood flow during LCx cannulation, resulting in profound myocardial ischemia, was considered the most likely mechanism. The procedure was aborted. After stabilization, the conscious patient was returned to the ICCU for continued monitoring and reconsideration of the revascularization strategy.

Approximately 12 h after the aborted procedure, the Heart Team reassessed the case. The combination of clinical and anatomical risk (EuroSCORE II 19.34%, SYNTAX score 25, and BCIS-CHIP score 12), the preceding cardiac arrest, and the need to treat the critically diseased left coronary system supported planned temporary MCS. IABP was considered to provide a lower level of hemodynamic support than required for the planned intervention because its effect depends on residual native cardiac function, and it does not provide active LV-to-aortic forward flow. Conversely, VA-ECMO would have represented a substantially more invasive form of cardiopulmonary support in a patient who was hemodynamically stable and not in cardiogenic shock before the procedure; moreover, peripheral VA-ECMO may increase LV afterload and potentially necessitate additional LV unloading. VA-ECMO was also unavailable at the time because all institutional systems were in use, while Impella was not available at our institution. iVAC2L was therefore selected to provide temporary active pulsatile LV-to-aortic support during the planned HR-PCI.

Immediately before the intervention, the patient was hemodynamically stable, with a blood pressure of 110/72 mmHg and a heart rate of 90 beats/min. Peripheral oxygen saturation was 90% while receiving oxygen at 13 L/min. No vasopressor or inotropic support was required. The total procedure duration was 130 min, including approximately 30 min for device preparation and management and 60 min of active pump support, and 180 mL of contrast was administered. Anticoagulation was achieved with 5000 IU of unfractionated heparin; the patient received antiplatelet therapy with aspirin and ticagrelor, and only local anesthesia with procaine was used. The iVAC2L was introduced through the right femoral artery using an 18 Fr introducer, while the PCI guiding catheter was advanced through the right radial artery. Before iVAC2L insertion, the right femoral artery and access site were assessed by ultrasound to evaluate the feasibility of large-bore arterial access. Computed tomography angiography was not performed to avoid additional iodinated contrast exposure, particularly given the patient’s stage 3 chronic kidney disease and the anticipated contrast requirement for complex PCI. Ultrasound assessment did not identify clinically significant peripheral arterial disease that would preclude 18 Fr femoral access. The system was operated at a setting corresponding to nominal pulsatile support of up to 2 L/min. A T and small protrusion (TAP) technique was used for bifurcation stenting. Before stent implantation, the lesions were predilated using balloons with diameters corresponding to the nominal diameters of the planned stents and the size of the respective vessel segments. Following stent implantation, proximal optimization technique (POT) was performed using a 5.0 mm balloon selected according to the larger reference diameter of the proximal main vessel rather than the nominal stent diameter in the distal segment. This approach was used to optimize expansion and apposition of the proximal stent segment while accounting for the diameter mismatch between the proximal and distal segments of the bifurcation. Given the urgent nature of the intervention, the repeat procedure was performed with the aim of restoring coronary flow while minimizing additional instrumentation (IVUS and OCT) and procedural duration. Due to the critical disease involving the LMCA, LAD, and LCx, a culprit-only strategy was considered inappropriate because it would have left other critical lesions untreated. In the context of the extensive left-coronary disease, the hemodynamic instability encountered during the previous intervention, and iVAC2L support already in place, LMCA, LAD, and LCx revascularization was therefore completed during the same procedure. Throughout the supported procedure, the patient remained conscious, breathed spontaneously, and remained hemodynamically stable without vasopressors. No recurrent ventricular tachycardia, ventricular fibrillation, or pulseless electrical activity was documented.

**Figure 3:**
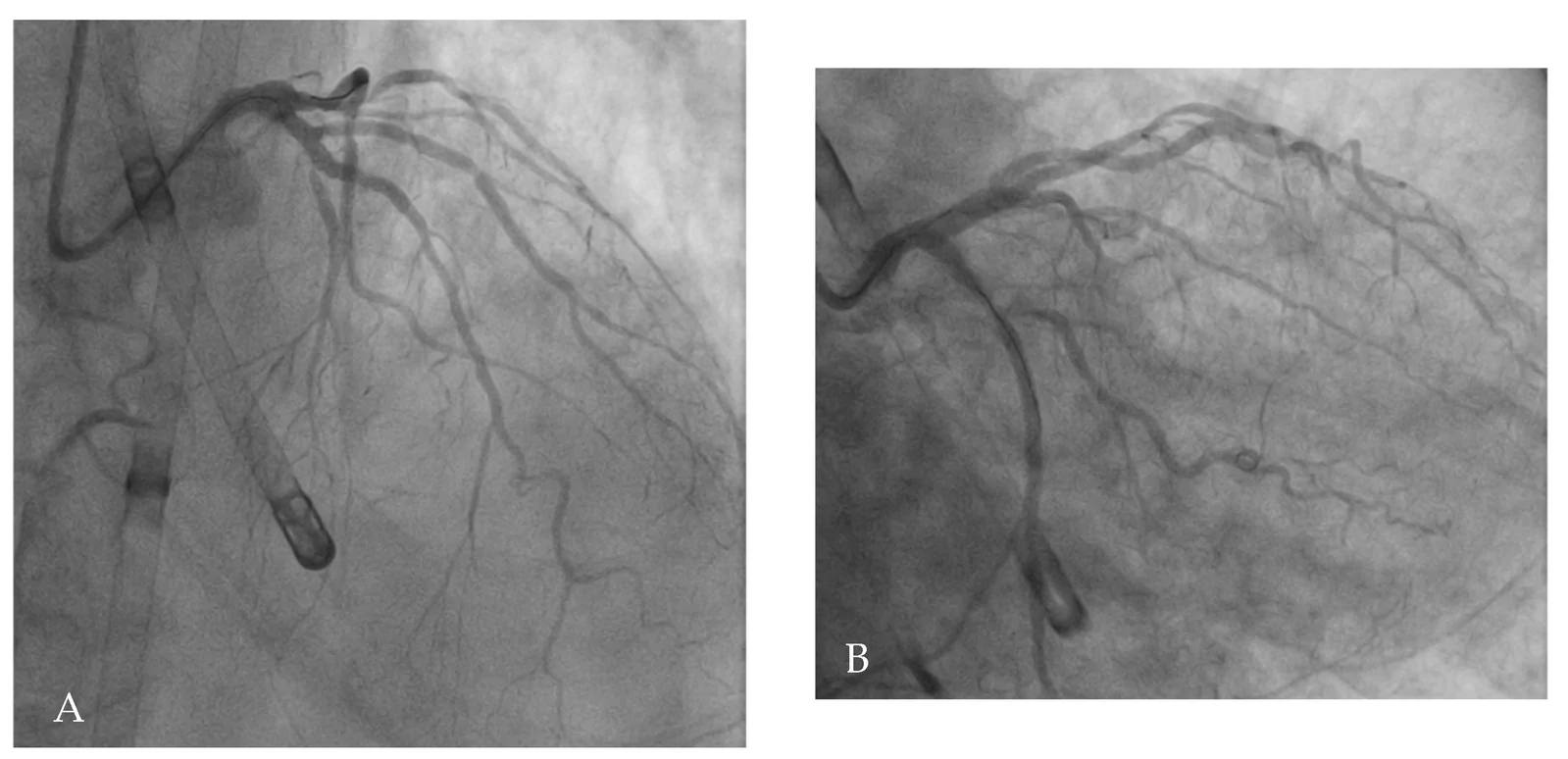
Final angiographic result after iVAC2L-supported HR-PCI. Panels (**A**,**B**) show the treated LMCA bifurcation, LAD, and LCx in two projections. The LAD continues anteriorly and distally from the LMCA bifurcation, whereas the LCx curves laterally in the atrioventricular groove. The iVAC2L catheter is positioned across the aortic valve, with its inlet in the left ventricle and outlet in the ascending aorta.

Five stents were implanted in the LMCA, LAD, and LCx, completing the planned left-coronary revascularization with final TIMI grade 3 flow (Figure 3). The right femoral access site was closed using a Perclose ProGlide device and an 8 Fr Angio-Seal closure device. Standardized postprocedural hemolysis and access-site outcome measures were not available. 

After the procedure, respiratory failure regressed, chest pain did not recur, and no other early complication was documented. One day later, the patient was transferred to the general cardiology ward. At discharge after a six-day hospital stay, repeat echocardiography showed a modest increase in LV ejection fraction to approximately 20%. He was discharged to an inpatient rehabilitation facility. At the four-month follow-up, the patient had no rehospitalizations, recurrent myocardial infarction, stroke, episodes of heart failure decompensation, or bleeding events. The key clinical events, interventions, and follow-up are summarized in Table 2.

## 3. Discussion

This case adds to the limited clinical literature on iVAC2L-supported HR-PCI. The principal observation is that, after an initial unsupported PCI attempt was complicated by cardiac arrest, the planned left-coronary intervention was completed during a second procedure performed with pulsatile MCS. No vasopressors were required, and no recurrent electrical instability was documented during the supported procedure. These findings suggest technical feasibility in one selected patient, but they do not establish that iVAC2L caused the favorable procedural or short-term clinical course. This case does not support routine use or superiority over other MCS devices.

The physiological rationale for planned MCS in this case was particularly strong because of the combination of severely impaired LV systolic function (LVEF 15%), critical LMCA, and multivessel disease, further emphasized by cardiac arrest during the initial unsupported PCI attempt. In this setting, transient reduction in coronary flow during repeat complex LMCA, LAD, and LCx PCI could be poorly tolerated, and temporary MCS was intended to provide additional circulatory reserve during the intervention rather than to treat established cardiogenic shock. Compared with previously published iVAC2L reports, the novelty of this case lies not in the use of the device itself, but in the dynamic reassessment of procedural risk and the convergence of acute STEMI, critical LMCA and multivessel disease, severe LV dysfunction, preceding procedure-related cardiac arrest, and the need for extensive repeat revascularization. Importantly, repeat Heart Team assessment, procedural replanning, greater preparedness for hemodynamic deterioration, and careful catheter manipulation may also have contributed.

Available physiological studies suggest that iVAC2L may provide additional hemodynamic support and reduce LV workload during HR-PCI, although clinical evidence remains limited. Pressure-volume data from the PULSE study suggest a potential reduction in LV workload and myocardial oxygen demand [8]. In the multicenter iVAC2L registry of 293 patients, device activation was associated with significant increases in systolic and diastolic blood pressure, cardiac output, and cardiac power output, without a significant change in heart rate [9]. However, pulmonary hemodynamic monitoring was available in only 50 patients, and unmeasured confounders, including intravenous fluid administration and the effects of revascularization itself, could have contributed to these changes. In a prospective comparison of iVAC2L and Impella 2.5, both devices were associated with increased aortic pressure, although the response developed more gradually with iVAC2L, while Impella 2.5 generated significantly greater additional flow [4]. A retrospective comparison of iVAC2L and IABP in HR-PCI found no significant differences in six-month mortality, repeat revascularization, or stroke, further emphasizing the need for larger prospective comparative studies [10]. However, results remain limited by small cohorts, heterogeneous indications, variable loading conditions, and limited comparative outcome data.

Device selection was individualized according to the anticipated hemodynamic support requirement, patient characteristics, and institutional availability. Given the severely impaired LV systolic function (LVEF 15%), critical LMCA and multivessel disease, and previous bradycardia/asystole during the initial PCI attempt, IABP was considered insufficient for the anticipated support requirement. Although VA-ECMO can provide greater systemic support, it was unavailable at the time, and, in a patient without cardiogenic shock, its greater invasiveness and potential to increase LV afterload were considered disproportionate to the intended prophylactic support strategy [11].

This case also emphasizes the importance of a structured protected-PCI pathway, including a predefined access plan, anticoagulation target, hemodynamic monitoring, support duration and weaning criteria, vascular-closure strategy, and surveillance for bleeding, limb ischemia, acute kidney injury, neurological events, and hemolysis. Intracoronary imaging may further facilitate optimization of complex stent deployment when clinically feasible and may reduce ischemic events [12,13].

The application of temporary MCS extends beyond HR-PCI to other high-risk interventional settings in cardiology. In patients with refractory ventricular arrhythmias undergoing catheter ablation, MCS may provide hemodynamic support during unstable ventricular tachycardia, facilitating mapping and ablation when the arrhythmia would otherwise be poorly tolerated. In this setting, as in HR-PCI, appropriate patient selection, a multidisciplinary approach, and timing of support remain important considerations [14].

This is a retrospective single-patient report from one center, without a control group or comparison with another MCS strategy. Continuous quantitative hemodynamic measurements were not available, so the magnitude of circulatory support cannot be objectively determined. The contribution of iVAC2L cannot be separated from the effects of revascularization, pharmacological treatment, operator decisions, and intensive care. An important limitation is the absence of intracoronary imaging; IVUS or OCT could have provided additional information for lesion assessment and optimization of stent expansion and apposition, particularly during complex LMCA bifurcation PCI. Follow-up was limited to four months. Standardized vascular, bleeding, renal, neurological, and hemolysis outcomes were not recorded, and detailed device and PCI consumable data were unavailable for this report. The increase in visually estimated LVEF from 10–15% to approximately 20% may reflect a combination of myocardial recovery, altered loading conditions, and measurement variability. Accordingly, the case should be interpreted as hypothesis-generating and should not be generalized to all patients undergoing HR-PCI.

Future prospective studies should evaluate standardized criteria for selecting patients for iVAC2L-supported HR-PCI and should incorporate continuous invasive hemodynamic monitoring, prespecified vascular and bleeding endpoints, renal function and hemolysis assessment, and longer clinical follow-up. Comparative studies against unsupported PCI and other temporary MCS strategies are needed to determine whether pulsatile support provides clinically meaningful benefit in specific anatomical or hemodynamic subgroups. Multicenter registries using uniform definitions of procedural success and adverse events could provide important safety information while adequately powered comparative trials are developed.

## 4. Conclusions

This case illustrates the technical feasibility of iVAC2L-supported HR-PCI in an older patient with severe LV systolic dysfunction and complex multivessel coronary disease after an unsupported PCI attempt was complicated by cardiac arrest. During the supported intervention, the planned left-coronary revascularization was completed without vasopressors or documented recurrent electrical instability, and the patient remained conscious and hemodynamically stable. Because this is a single case without continuous quantitative hemodynamic measurements or a comparator, no conclusion regarding comparative safety or efficacy can be drawn. Prospective studies are required to define patient selection and clinically meaningful outcomes.

## Figures and Tables

**Table 1:** Dynamics of laboratory parameters. HBG, hemoglobin; RBC, red blood cell; PLTs, platelets; WBC, white blood cell; CREA, creatinine; K^+^, potassium; LAC, lactate; CRP, C-reactive protein; hs-cTnI, high-sensitivity cardiac troponin I; TnI, troponin I; NT-proBNP, N-terminal pro-B-type natriuretic peptide; NM, not measured.

	Day	Day 1 Before Initial PCI	Day 2 Before Second PCI	Day 3	Day 4	Day 5	Discharge Day
Lab. Parameters							
HGB (g/L)		125	NM	115	NM	NM	113
RBC (10^12^/L)		4.28	NM	4.19	NM	NM	4.17
PLT (10^9^/L)		259	NM	241	NM	NM	239
WBC (10^9^/L)		10.41	NM	10.88	NM	NM	10.76
CREAT (μmol/L)		93	NM	115	NM	NM	101
K^+^ (mmol/L)		3.28	NM	3.81	NM	NM	4.36
LAC (mmol/L)		3	NM	NM	NM	NM	NM
CRP (mg/L)		41	NM	46	NM	NM	34
hs-cTnI (ng/L)		2905	NM	NM	NM	NM	NM
TnI (ng/L)		NM	NM	908.4	NM	NM	NM
NT-proBNP (ng/L)		6324	NM	NM	NM	NM	8839

**Table 2:** Timeline of presentation, clinical deterioration, revascularization attempts, iVAC2L-supported HR-PCI, and follow-up. STEMI, ST-segment-elevation myocardial infarction; hs-cTnI, high-sensitivity cardiac troponin I; CAG, coronary angiography; RCA, right coronary artery; LMCA, left main coronary artery; LAD, left anterior descending artery; LCx, left circumflex artery; HR-PCI, high-risk percutaneous coronary intervention.

Clinical Stage	Key Events
Admission	Inferior STEMI, hs-cTnl 2905 ng/L
Initial CAG and PCI	RCO chronic total occlusion. LMCA 99%, LAD 99%, LCx 70%. Cardiac arrest
Clinical deterioration	Pulmonary edema, recurrent chest pain, ventricular tachycardia
Heart team	Surgical revascularization declined. Decision: iVAC2L-supported HR-PCI
HR-PCI	Five stents were implanted in the LMCA, LAD, and LCx, completing the planned left-coronary revascularization with final TIMI grade 3 flow
Outcome	Hemodynamically stable. Discharge day 6. 1- and 4- month follow up: no major cardiovascular events

## Data Availability

All data relevant to this case are included in the article. Additional individual-level clinical data are not publicly available because of patient privacy.

## Supplementary Materials

The following supporting information can be downloaded at: https://www.mdpi.com/article/10.3390/jcm15187322/s1, File S1. CARE Checklist of information to include when writing a case report.
